# Mortality Trend of Cerebrovascular Disorders: A Cross-sectional Study in Northern Iran (2016—2023)

**DOI:** 10.34172/aim.34939

**Published:** 2026-02-01

**Authors:** Amirhossein Alizadeh-Nodehi, Mohammad–Ali Jahani, Pouyan Ebrahimi, Amir-Hossein Lashkarbolouki, Amin Esmaeilnia Shirvani, Ali Ramzaninezhad, Hossein-Ali Nikbakht

**Affiliations:** ^1^Department of Epidemiology and Biostatistics, School of Public Health, Tehran University of Medical Sciences, Tehran, Iran; ^2^Social Determinants of Health Research Center, Health Research Institute, Babol University of Medical Sciences, Babol, Iran; ^3^Student Research Committee, Babol University of Medical Sciences, Babol, Iran; ^4^School of Medicine, Babol University of Medical Sciences, Babol, Iran

**Keywords:** Cerebrovascular disorders, Iran, Joinpoint regression, Stroke

## Abstract

**Introduction:**

Cerebrovascular disorders (CeVDs) are among the leading causes of death worldwide. This study aims to analyze the trends in mortality due to CeVDs in northern Iran between 2016 and 2023.

**Methods:**

In this cross-sectional study, we included all documented Deaths due to CeVDs in Babol County, north of Iran, from 2016 to 2023. The crude mortality rate (CMR) and age-standardized mortality rate (ASMR) were used to assess the trends in CeVDs mortality. The Cochran-Armitage trend test and Joinpoint regression were employed to analyze the changes in disease trends.

**Results:**

During the study period, a total of 1,798 deaths from CeVDs were reported, with a mean age of 76.8±13.2 years. The CMR and ASMR for CeVDs decreased from 38.4 and 28.3 per 100,000 population in 2016 to 30.9 and 23.3 per 100,000 in 2023 (*P*<0.001). The trend decreased in both genders and urban and rural populations (*P*<0.05). Joinpoint regression indicated a break point in 2018. Between 2018 and 2023, the ASMR trend decreased, with an annual percentage change of -7.98% (95% CI, -18.72% to -4.88%).

**Conclusion:**

This study reveals a significant decrease in CeVDs mortality in the region, indicating progress in therapeutic interventions. However, death rates in rural areas remain high. Addressing this condition requires targeted approaches and increased resource allocation.

## Introduction

 Cardiovascular diseases (CVDs) are the leading global cause of mortality and represent a complex public health issue.^1,2^ In 2021, these diseases were responsible for 20.5 million deaths and 422 million years of life lost due to disability and death (DALY).^3^ Strokes, ranking as the second most prevalent form of CVDs, were recognized as the third leading contributor to global mortality and the fourth major factor in years of life lost (YLL) due to fatality and disability in 2021.^4,5^ Additionally, strokes directly and indirectly impose a global cost of approximately $892 billion, accounting for about 1.2% of the world’s GDP.^6^ It is projected that by 2050, strokes will become the second most common cause of death and the leading cause of years of life lost.^2^ Furthermore, these diseases are expected to cause 12.5 million fatalities and 224.86 million DALYs lost due to mortality and morbidity by the year 2050.^7^

 The crude mortality rate (CMR) due to Cerebrovascular Disorders (CeVDs) has shown varying trends across regions and populations.^8^ In high-income countries, the mortality rate due to stroke has generally decreased over the past few decades.^8,9^ Between 1980 and 2016, most European countries experienced a reduction in CeVDs mortality.^10^ In Japan, the mortality rate from CeVDs decreased in men and women from 1995 to 2019. However, the trend for mortality due to intracerebral hemorrhage remained relatively stable during these years.^11^ In 2019, the mortality rate and DALY from stroke in low-income countries were 3.6 and 3.7 times higher, respectively, than in high-income countries.^8^ In low-income countries, a higher proportion of individuals experience hemorrhagic strokes and tend to die from strokes at younger ages.^9,12^ A study conducted in the Zanzibar region of Ethiopia showed that the age of stroke onset in this area was lower compared to Western countries. It was also found that the age-standardized incidence rate (ASIR) of stroke and the frequency of brain hemorrhages in this region were higher than in high-income countries.^13,14^

 In Iran, in 2019, the age-standardized mortality rate (ASMR) and DALY from stroke were lower than the global average, as well as the averages for the Middle East and North Africa. Although the ASIR of strokes in Iran was lower than the average for the Middle East and North Africa, it was higher than the global average.^15^ The ASMR has shown a decreasing trend in Iran from 1990 to 2019, and the increase in new stroke cases and the number of stroke-related deaths in these years was primarily due to population growth.^16^ Understanding the trends in mortality related to CeVDs is crucial for public health planning and the formulation of intervention strategies.^17^ This study aims to examine the trend in mortality due to CeVDs from 2016 to 2023 in Babol County to guide public health policies and interventions to maintain, improve, and enhance the population’s health.

## Materials and Methods

 This retrospective cross-sectional study examined the mortality trend due to CeVDs in Babol, the second most populous county in northern Iran, over eight years from 2016 to 2023. The study population consisted of all registered deaths due to CeVDs in Babol, recorded in the mortality cause classification system of the Health Department of Babol University of Medical Sciences from 2016 to 2023. The sampling method was a census of all recorded death cases. The data used in this system was sourced from reliable institutions, including cemeteries, forensic medicine, hospitals, and experienced physicians involved in death-cause registration. The records were examined for duplicate entries to ensure the integrity of mortality cause data, and the dataset variables were validated through cross-referencing with other documented information. Causes of death with unlikely codes based on gender and age, those with improbable fatal outcomes, and poorly defined or nonsensical cause codes were examined and corrected. In cases where the cause of death appeared unlikely or rare for a given gender or age, the relevant information was requested from the source, and corrections were made by experts reviewing the appropriate records. After eliminating duplicates and redistributing improbable or poorly defined codes, program officials finalized and validated the data at the Ministry of Health, ensuring reliability for reporting purposes.

 The cause of death was coded in accordance with the 10th edition of the International Classification of Diseases and Mortality (ICD-10). CeVDs belong to the broader category of CVDs and are assigned 10 codes (I60-I69). These CeVDs codes include I60 (Subarachnoid hemorrhage), I61 (Intracerebral hemorrhage), I62 (Other non-traumatic intracranial hemorrhage), I63 (Cerebral infarction), I64 (Stroke, not specified as hemorrhage or infarction), I65 (Occlusion and stenosis of precerebral arteries, not resulting in cerebral infarction), I66 (Occlusion and stenosis of cerebral arteries, not resulting in cerebral infarction), I67 (Other cerebrovascular diseases), I68 (Cerebrovascular disorders in diseases classified elsewhere), and I69 (Sequelae of cerebrovascular disease).^18^

 To calculate and compare the CMR due to CeVDs, it was essential to estimate the population of the district from 2016 to 2023. Accordingly, the mean annual population growth rate was determined utilizing the following formula, derived from the census data for each year: $\left\{r=\sqrt[{n}]{p(n)/p(0)}-1\right\}$ . In this formula, ***r*** represents the annual growth rate. The term *p*(*n*) denotes the population in year ***n***, referring to the target year’s population. Similarly, *p*(0) indicates the population in the initial year (year zero). The variable ***n*** defines the number of years between the initial and target years.

 Descriptive statistics were presented as the mean and standard deviation for continuous variables, while categorical variables were summarized using frequency and percentage. We also provided 95% confidence intervals for CMR and ASMRs in this study. A CMR was calculated using the National Statistical Center of Iran’s census information on Babol County population, classified by gender, Residential Status, and age. The direct standardization method was employed to compute the ASMR, utilizing the Segi standard population.^19^ A Cochran-Armitage trend test was used to analyze mortality trends over the specified period. Data were analyzed using SPSS (version 22) and STATA (version 14), with *P* < 0.05 considered statistically significant. GraphPad Prism (version 8) was also used to plot study-related charts.

 To examine changes in the trend of age-standardized mortality rates (ASMR) for cerebrovascular disorders over the study period, we applied Joinpoint regression analysis using the Joinpoint Regression Program version 5.3.0, a specialized tool for trend analysis in public health research. The dependent variable was the Age-Standardized Mortality Rate (ASMR) for cerebrovascular disorders, and years of event served as the sole predictor variable.

 To model the relationship between ASMR and time, a log link function was applied. By default, the Joinpoint regression software uses a log transformation of the dependent variable. This transformation impacts both the fit and the interpretation of the trend model. Selecting a log transformation fits a linear model to the trend on a log scale. This option provides output that allows us to directly compare relative differences and annual percent changes (APCs) during the period. Additionally, we choose to apply the log transformation based on the type of outcome (rates, counts, or percentages) to improve interpretability or ease of reporting the results.^20,21^

 In this study, it was assumed that the random errors are heteroscedastic. The Joinpoint software automatically calculates the standard error (SE) under the “Standard Error (Calculated)” option, which is the default setting of the program. With this option, Weighted Least Squares (WLS) is applied to estimate the regression coefficients, ensuring the model appropriately handles heteroscedasticity, providing more reliable and efficient estimates.^22^

 Model fitting was performed using the Uncorrelated errors option, which is recommended in the Joinpoint software documentation. This specification was selected based on the presence of negative autocorrelation in the residuals, a typical feature in time-series data. The uncorrelated errors model effectively addresses this issue by avoiding overfitting to noise and ensuring more stable estimates. While autocorrelation can be estimated and incorporated into the model, doing so may reduce the power to detect Joinpoints. In our analysis, models with autocorrelation adjustments yielded results similar to the uncorrelated model, reinforcing the validity of the selected approach. The estimated autocorrelation parameter for all groups confirmed the presence of negative autocorrelation, further supporting the decision to use the uncorrelated errors specification.^22^

 The final model was selected using the Weighted Bayesian Information Criterion (Weighted BIC), which is preferred in Joinpoint regression when the goal is to identify a model that performs reliably across a wide range of scenarios. Compared to other model selection criteria, such as BIC, Permutation Test, and BIC3, the Weighted BIC is more robust and adaptable, making it the optimal choice for diverse data conditions.^23^

## Results

**Figure 1:**
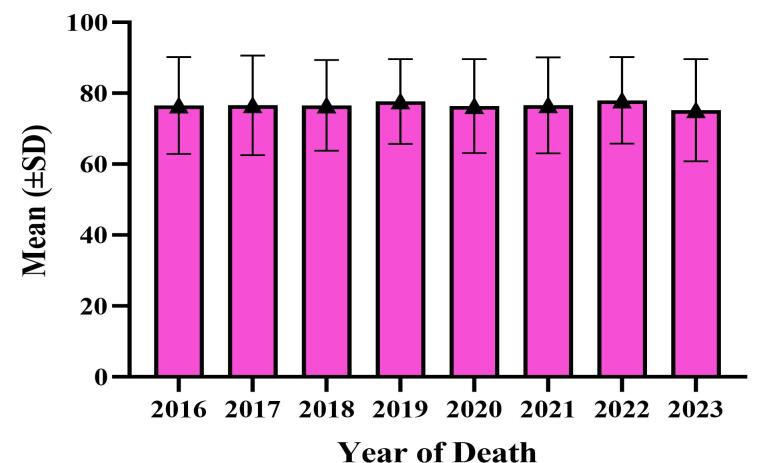
Mean (standard deviation) Age of Cerebrovascular Disorders Deaths by the Studied Years in the in Babol County (2016–2023)

 During the study period from 2016 to 2023, a total of 1,798 deaths due to CeVDs were reported, of which 906 cases (50.4%) were female and 1,038 cases (57.7%) were from rural areas. The mean age of deceased individuals during the study period was 76.8 ± 13.2 years, with an age range of 9 to 106 years. The mean age of deaths, stratified by study years, is presented in Figure 1. The lowest mean age was observed in 2023 (75.2 ± 14.4 years), while the highest mean age was recorded in 2022 (78.0 ± 12.2 years). However, no significant differences were observed in the mean age across the study years.

**Figure 2:**
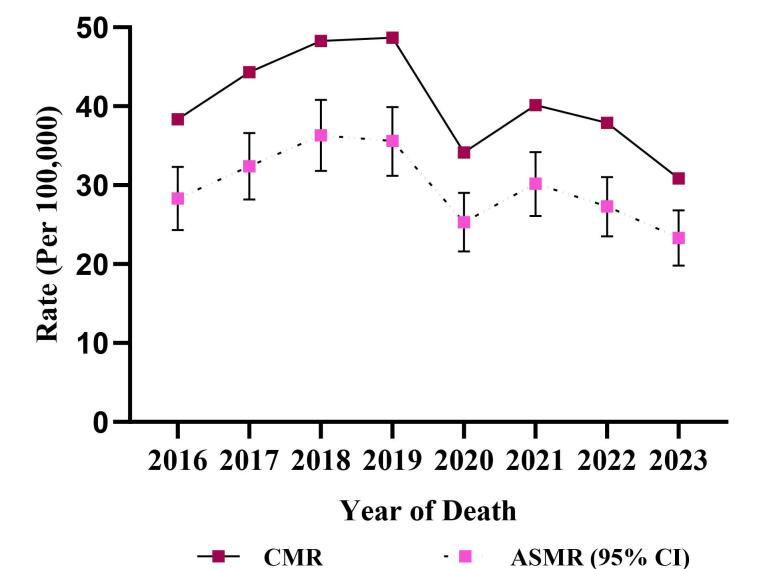
Crude and Age-standardized Trends in the Mortality Rate (CMR and ASMR) of Cerebrovascular Disorders Per Hundred Thousand People in Babol County (2016–2023). The Error Bars Indicate the 95% Confidence Interval for ASMR

 The CMR was consistently higher than the ASMR for CeVDs across all years (Figure 2). The highest CMR and ASMRs were observed in 2019, at 48.7 and 35.6 per 100,000 population, respectively, while the lowest rates were recorded in 2023, at 30.9 and 23.3 per 100,000 population. At the beginning of the study period in 2016, the CMR and ASMRs for CeVDs were 38.4 and 28.3 per 100,000 population, respectively. These rates declined throughout the study period, reaching 30.9 and 23.3 per 100,000 in 2023, a statistically significant reduction (*P* < 0.001).

**Figure 3:**
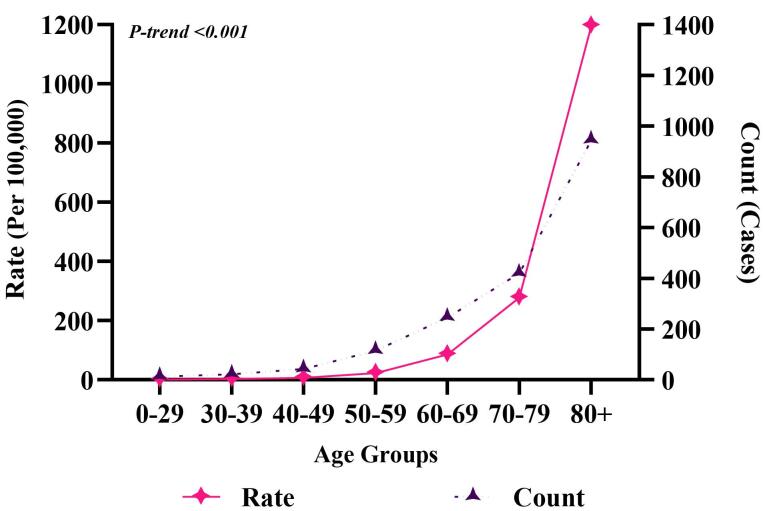
Examining the Trend, the Number of Deaths, and the Mortality Rate Per Hundred Thousand Population due to the Cerebrovascular Disorders according to age groups in Babol County (2016–2023)

 A CMR of 0.6 per 100,000 population was observed in the 0–29 age group for CeVDs. CeVDs CMR in this age group was 0.6 per 100,000 in males and females (although the mortality rate of cerebrovascular diseases was 0.61 in men and 0.62 in women in the 0–29 age group, they became equal due to rounding). Also, CeVDs CMR in rural areas (1.1 per 100,000) were higher than those in urban areas (0.3 per 100,000). Furthermore, elderly individuals aged 80 and above had the highest CMR, reaching 1,196.6 per 100,000. Among the elderly, the mortality rate of CeVDs was 1,209.8 per 100,000 for males, 1,183.3 per 100,000 for females, 877.4 per 100,000 for urban areas, and 1,628.5 per 100,000 for rural areas. A continuous increase in the number and mortality rate of CeVDs was observed with advancing age, as well as across both genders and among residents of both urban and rural areas. This upward trend was statistically significant in the overall pattern and across all four subgroups (*P* < 0.001) (Supplementary file, Figures S1 and S2) (Figure 3).

 The CMR of CeVDs was consistently higher in rural areas than urban areas throughout the study period. The highest CMR and ASMRs among females and rural residents were recorded in 2018, whereas for males and urban residents, the peak mortality rate was observed in 2019. The lowest CMR for CeVDs among males was reported in 2020, while for females, urban residents, and rural residents, the lowest rates were recorded in 2023. The mortality trend for CeVDs showed a statistically significant decline across all four subgroups mentioned (*P* < 0.001) (Table 1).

**Table 1:** Crude and Age-standardized Mortality Rates of Cerebrovascular Disorders by the Studied Years, Gender and Residential Status Per Hundred Thousand Population in Babol County (2016–2023).

**Years**	**Male**			**Female**			**Urban**			**Rural**		
	**Crude Mortality rate**	**Age-standardized Mortality Rate**		**Crude Mortality rate**	**Age-standardized Mortality Rate**		**Crude Mortality rate**	**Age-standardized Mortality Rate**		**Crude Mortality rate**	**Age-standardized Mortality Rate**	
		**Rate**	**95% Confidence Interval**		**Rate**	**95% Confidence Interval**		**Rate**	**95% Confidence Interval**		**Rate**	**95% Confidence Interval**
2016	38.8	28.2	22.6-33.8	37.9	28.4	22.7-34.2	26.8	19.8	15.3-24.2	54.0	39.8	32.5-47.1
2017	43.4	30.8	25.1-36.6	45.2	34.0	27.7-40.3	34.5	24.9	20.1-29.8	57.6	42.5	35.0-50.0
2018	44.6	33.4	27.3-39.5	52.0	39.3	32.6-46.0	29.9	22.3	17.6-27.0	73.1	55.3	46.7-63.8
2019	50.1	36.5	30.3-42.8	47.3	34.6	28.5-40.7	39.5	29.6	24.3-34.9	61.1	43.6	36.3-50.9
2020	30.0	21.9	17.1-26.7	38.4	28.7	23.1-34.3	25.7	18.3	14.2-22.4	45.6	34.7	28.0-41.4
2021	44.9	33.6	27.6-39.6	35.4	26.6	21.2-32.0	31.1	23.6	18.8-28.4	52.4	39.0	32.0-46.1
2022	35.3	25.2	20.2-30.3	40.4	29.3	23.8-34.8	26.2	19.2	15.1-23.4	53.7	38.1	31.4-44.8
2023	30.5	22.7	17.8-27.5	31.3	23.9	18.8-29.0	23.4	17.8	13.7-21.8	40.9	30.8	24.6-36.9
P-trend	0.013^*^			0.008^*^			0.047^*^			0.002^*^		

^*^Significant at *P* < 0.05

 The analysis of ASMRs for CeVDs in Babol County from 2016 to 2023, using the Joinpoint regression model, identified a Joinpoint in 2018, leading to the formation of two distinct periods (Supplementary file, Figure S3). Between 2016 and 2018, the ASMR exhibited an increasing trend, with an annual percentage change (APC) of 13.00% (95% CI, -0.84% to 30.46%). However, this upward trend was not statistically significant. Meanwhile, the ASMR decreased statistically significantly from 2018 to 2023, with a proportional change of -7.98% (95% CI, -18.72% to -4.88%). The Joinpoint regression model did not detect any Joinpoints for males and urban residents. Although the ASMR for these two groups followed a declining trend, the reduction was not statistically significant. Similar to the overall pattern, the model identified a Joinpoint in 2018 for females and rural residents, dividing the timeline into two periods. The ASMR for females increased significantly between 2016 and 2018, with an APC of 14.47% (95% CI, 1.04% to 31.52%). However, the observed increase during this period was not statistically significant for rural residents (Table 2).

**Table 2:** Trend of Cerebrovascular Disorders Age-Standardized Mortality Rate in Babol County by Gender and Residential Status.

	**Trend 1**		**Trend 2**		**AAPC**^b^** % (95% CI) (2016-2023**)
**Variable**	**APC**^a^** % (95% CI)**	**Years**	**APC % (95% CI)**	**Years**	
All Deaths	13.00 (-0.84 to 30.46)	2016-2018	-7.98* (-18.72 to -4.88)	2018-2023	-2.42 (-6.54 to 1.17)
Gender					
Male	-3.27 (-12.33 to 6.30)	2016-2023	—	—	-3.27 (-12.33 to 6.30)
Female	14.47* (1.04 to 31.52)	2016-2018	-8.60*(-16.20 to -5.81)	2018-2023	-2.53 (-6.25 to 0.82)
Residential Status					
Urban	-2.91 (-11.29 to 5.91)	2016-2023	—	—	-2.91 (-11.29 to 5.91)
Rural	13.51 (-1.23 to 31.69)	2016-2018	-8.86^*^ (-20.26 to -5.39)	2018-2023	-2.96 (-7.49 to 0.73)

a: Annual Percent Change, b: Average Annual Percent Change, ^*^significant at *P* < 0.05

## Discussion

 This study analyzed 1,798 deaths attributed to CeVDs in Babol County from 2016 to 2023. The mean age at death during the study period was 76.8 years, with no significant variations. Throughout the study period, the CMR and ASMR for CeVDs were consistently higher in rural areas compared to urban areas (Rural vs. Urban CMR: 54.0 vs. 26.8 in 2016 and 40.9 vs. 23.4 in 2023; ASMR: 39.8 vs. 19.8 in 2016 and 30.8 vs. 17.8 in 2023). Overall, CMR and ASMRs declined from 2016 to 2023, decreasing from 38.4 and 28.3 per 100,000 population in 2016 to 30.9 and 23.3 per 100,000 in 2023.

 When comparing CeVDs CMRs in our region with those in other parts of the world, it can be observed that the mortality rate in this region is lower than the global average. The World Stroke Organization estimated the global CMR for strokes in 2022 to be 84.69 per 100,000 population.^24^ This rate was reported to be 85.8 per 100,000 for men and 83.53 per 100,000 for women. A study analyzing global stroke mortality statistics up to 2022 identified Bulgaria as having the highest CMR, with 294 deaths per 100,000 people, followed by Latvia with 271 deaths per 100,000 people.^25^ According to the same study, Qatar had the lowest CMR in 2016, Honduras had the highest, with 4.7 deaths per 100,000 in 2013, and Bahrain had the highest, with 6.2 deaths per 100,000 in 2014.^26^

 The mortality trend of CVDs has shown a declining pattern in most regions worldwide, similar to ours. A study conducted in China, which examined stroke mortality trends from 2004 to 2019, reported a significant reduction of 39.8%, with ASMRs decreasing from 195 per 100,000 to 117.4 per 100,000 during the study period.^27^ Similarly, another study in Brazil demonstrated a decline in CeVDs mortality between 2000 and 2019.^28^ Consistent with these findings, a study conducted in Fars Province, Iran, demonstrated similar declining patterns. In this region, the standardized mortality rate significantly decreased from 45.4 to 29.7 per 100,000 population between 2004 and 2019.^29^ Possible explanations for this decline include advancements in specialized stroke management units, improved imaging technologies, and innovative treatment approaches. Intravenous thrombolytic agents such as alteplase and thrombectomy procedures are among these treatments, which can be effective if administered within six hours of ischemic stroke symptom onset.^30,31^ In our study, the trend of ASMRs significantly declined between 2018 and 2023, with an annual percentage change (APC) of -7.98%. Additionally, a statistically significant increase was observed among females between 2016 and 2018, followed by a significant decline from 2018 to 2023. A European study spanning 37 years (1980–2016) also reported a decreasing trend in CeVDs mortality, with a median annual percentage change of -2.7% for both men and women.^10^ Similarly, a study conducted in the United States (USA) showed a significant reduction in CeVDs deaths, with a relative percentage change (RPC) of -18% from 2006 to 2014.^32^

 According to our study, men and women do not die from CeVDs at significantly different rates. Previous studies have reported varying findings, with some indicating higher mortality in men, others showing higher mortality in women, and some reporting no significant gender-based differences.^27,29,33,34^ Consistent with our findings, a meta-analysis of stroke risk factors in Iran reported that stroke was not significantly associated with gender in this population.^35^ Our findings align with other studies demonstrating higher CeVDs CMRs in rural areas than urban regions.^27,36^ A primary reason for this disparity is the limited access to appropriate stroke treatment in rural areas. Since effective thrombolytic therapy requires administration within a critical “golden time window,” delayed arrival at well-equipped medical centers, which are predominantly located in urban areas, increases CMRs among rural patients. A study conducted in the USA between 2012 and 2017 analyzed over 700,000 stroke-related hospitalizations and reported that among patients with acute ischemic stroke, only 4.2% of rural patients received intravenous thrombolysis therapy, compared to 9.2% of urban patients.^37^ Similarly, access to endovascular treatment was also lower in rural settings, with CMRs increasing as the distance from urban centers increased. Beyond these factors, a higher incidence of stroke and a greater prevalence of associated risk factors in rural populations also contribute to the elevated CMRs observed in these areas.^38^ For instance, evidence from the neighboring Golestan Cohort Study identifies opium consumption, which is notably more prevalent in rural areas of Iran, as a critical risk factor for developing hemorrhagic strokes and increasing fatality rates in ischemic cases.^39,40^

 Throughout the study period, the mean age of stroke incidence remained in the seventh decade of life, which is consistent with results from other provinces in Iran.^41-43^ Although we observed the lowest mean age of stroke onset in 2023, the overall trend remained stable during the study years. In contrast, Wang *et al.* found that the mean age of stroke onset decreased by 0.28 years annually from 1992 to 2014 in a low-income rural population in China, primarily due to the increasing prevalence of stroke risk factors among younger adults.^44^ In our study, the CMR for CeVDs increased with age in both men and women, with the highest mortality rates observed in individuals aged 80 years and above, reaching 1,196.6 deaths per 100,000 population. A historical cohort study in the Khorasan Razavi Province (The northeast of Iran) found that for every one-year increase in patient age, the odds of in-hospital mortality rose by 7%.^45^ These findings underscore the role of age as a critical determinant of stroke risk and outcomes.^46^

 Our study was limited in that we lacked data on risk factors and treatments administered in each case of mortality. It could have provided insights into potential underlying causes and different treatment approaches for reducing mortality from CeVDs. Another limitation was the potential underreporting of actual mortality cases due to misdiagnosis or lack of registration in the health department’s database. Our study had the advantage of including a large number of mortality cases over an eight-year period. Consequently, demographic, gender-based, and urban-rural disparities could be thoroughly examined. Additionally, the application of Joinpoint regression significantly contributed to accurately mapping the annual mortality trends.

## Conclusion

 This study shows a significant reduction in CeVDs mortality in Babol County from 2016 to 2023. This aligns with declining mortality trends observed in various regions worldwide. These findings underscore the effectiveness of medical interventions in reducing mortality rates. However, death rates in rural areas remain high. Addressing this issue requires targeted strategies to allocate additional resources to rural regions. This would ensure early diagnosis and timely treatment of patients during the critical golden hour.

## Supplementary File

 Figure S1. Trends in Cerebrovascular Mortality by Age and Gender, Babol (2016–2023).
Figure S2. Trends in Cerebrovascular Mortality by Age and Residence, Babol (2016–2023).
Figure S3. Age-Standardized Cerebrovascular Mortality Trends, Babol (2016–2023).
