# Supplementary material for: Mortality Trend of Cerebrovascular Disorders: A Cross-sectional Study in Northern Iran (2016—2023)
Source: Arch Iran Med. 2026 Feb 1;29(2):62–8. doi: 10.34172/aim.34939 (PMC13477562; doi:10.34172/aim.34939)
Supplement: Supplementary file 1 [file aim-29-62-s001.pdf]

Arch Iran Med. February 2026;29(2):62-S1

doi:10.34172/aim.34939

http://journalaim.com

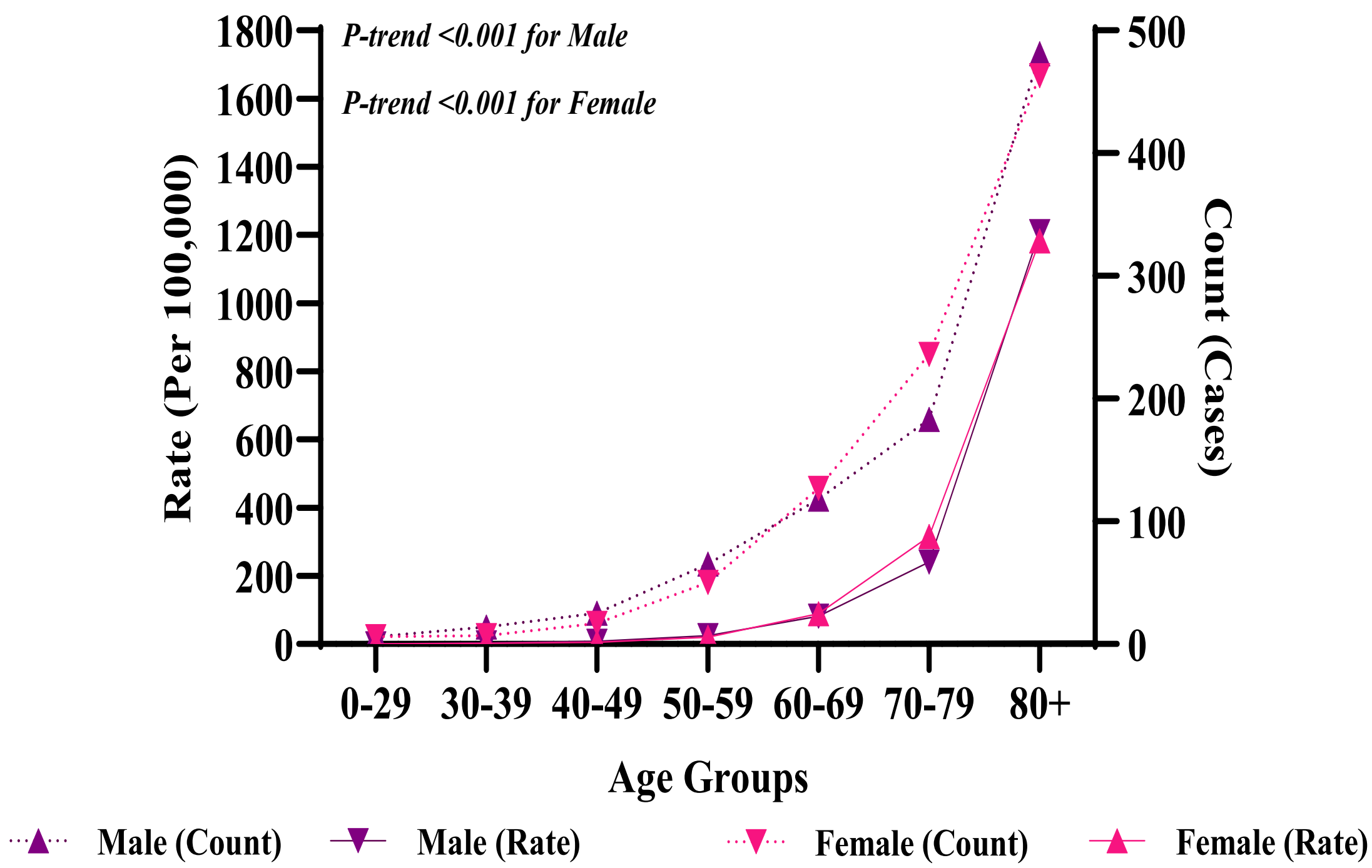

Figure S1. Examining the Trend, the Number of Deaths, and the Rate of Death Per Hundred Thousand Population Due to the Cerebrovascular Disorders According to Age Groups and Gender in Babol County (2016–2023).

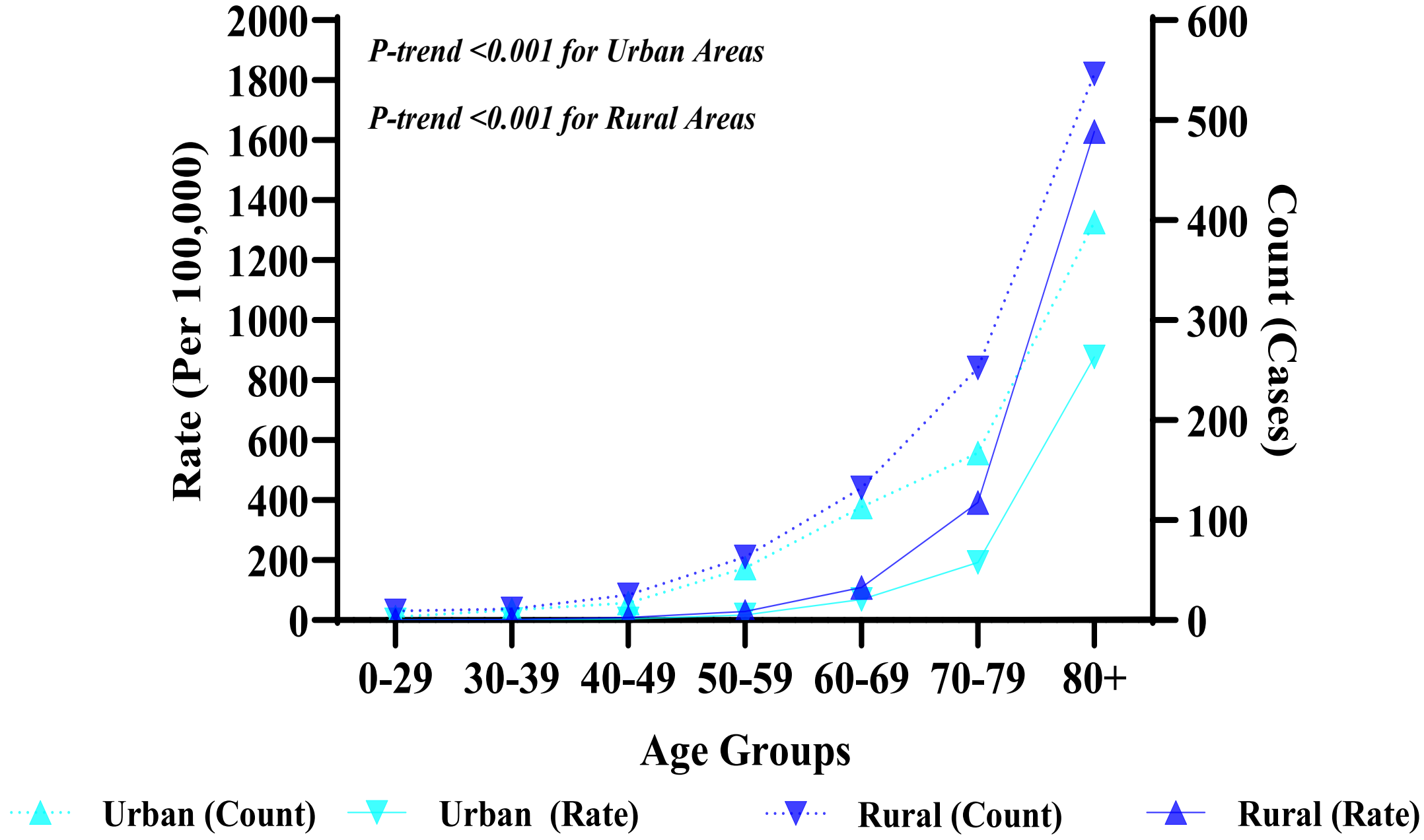

Figure S2. Examining the Trend, the Number of Deaths, and the Rate of Death Per Hundred Thousand Population Due to the Cerebrovascular Disorders According to Age Groups and Residential Status in Babol County (2016–2023).

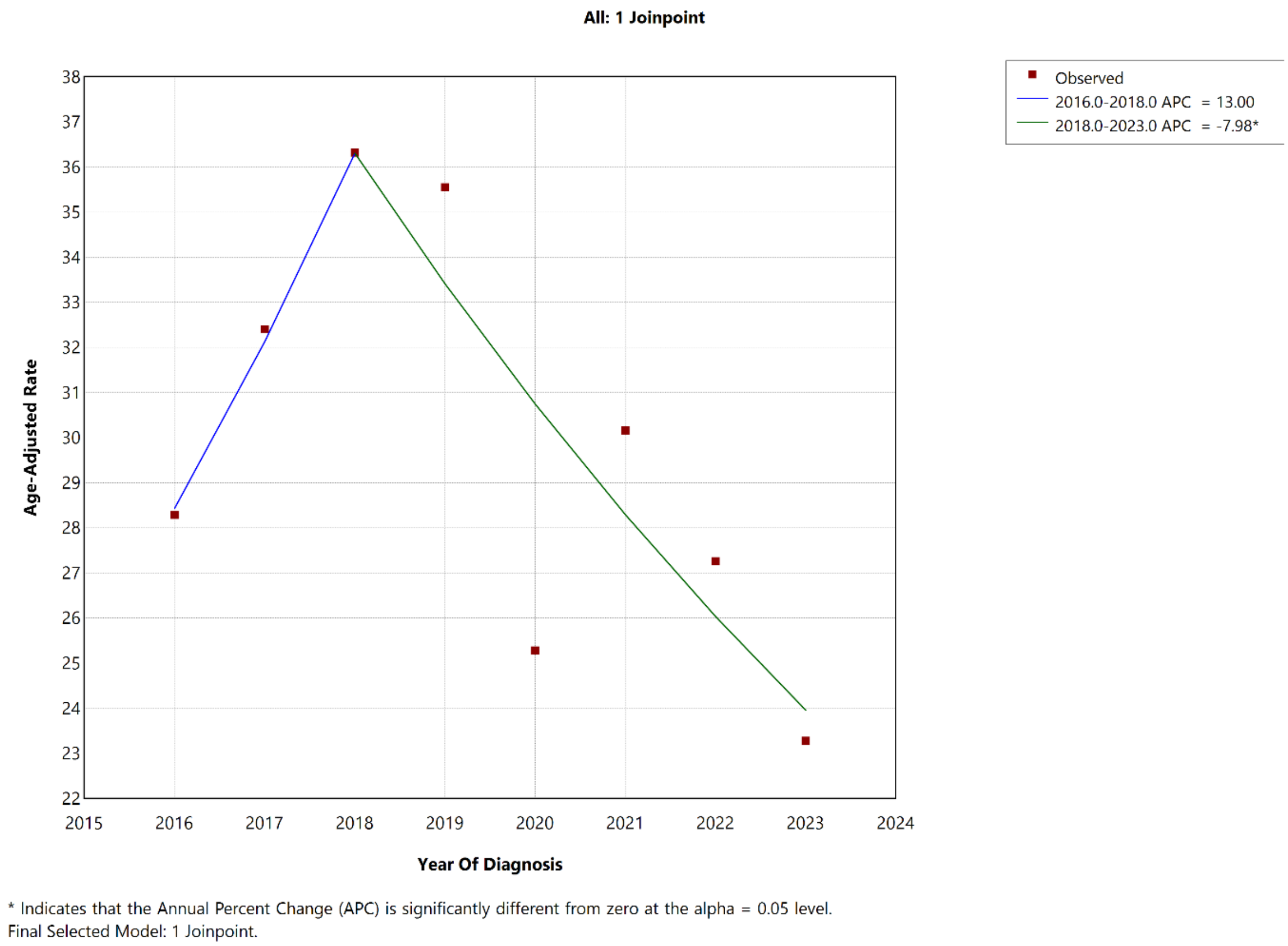

Figure S3. Trend of Cerebrovascular Disorders Age-Standardized Mortality Rate in Babol County During 2016-2023.
